# Population Divergence in Venom Bioactivities of Elapid Snake *Pseudonaja textilis*: Role of Procoagulant Proteins in Rapid Rodent Prey Incapacitation

**DOI:** 10.1371/journal.pone.0063988

**Published:** 2013-05-14

**Authors:** Jure Skejić, Wayne C. Hodgson

**Affiliations:** 1 Department of Biochemistry and Molecular Biology, BIO21 Institute, University of Melbourne, Victoria, Australia; 2 Monash Venom Group, Department of Pharmacology, Monash University, Clayton, Victoria, Australia; Universidade Federal do Rio de Janeiro, Brazil

## Abstract

This study looked at how toxic proteins in venoms of adult Australian eastern Brown snakes *Pseudonaja textilis* from South Australian and Queensland populations interact with physiological functions of the lab SD rat *Rattus norvegicus*. Circulatory collapse and incoagulable blood occurred instantly after injection of venom under the dorsal skin of anaesthetised and mechanically ventilated rats in an imitation of a *P. textilis* bite. Intravenous injection of purified *P. textilis* (Mackay, QLD) venom prothrombin activator proteins caused instant failure of circulation, testifying of high toxicity of these proteins and suggesting their role in rapid incapacitation of rodent prey. The hypothesis is further supported by circulatory collapse occurring instantly despite artificial respiration in envenomed rats and the finding of extremely high venom procoagulant potency in rat plasma. LC-MS and physiology assays revealed divergent venom composition and biological activity of South Australian (Barossa locality) and Queensland (Mackay locality) populations, which may be driven by selection for different prey. The Queensland venom of *P. textilis* was found to be more procoagulant and to exhibit predominately presynaptic neurotoxicity, while the South Australian venom contained diverse postsynaptic type II and III α-neurotoxins in addition to the presynaptic neurotoxins and caused significantly faster onset of neuromuscular blockade in the rat phrenic nerve-diaphragm preparation. LC-MS analysis found evidence of multiple coagulation factor X-like proteins in *P. textilis* venoms, including a match to *P. textilis* coagulation factor X isoform 2, previously known to be expressed only in the liver.

## Introduction

Several studies have found a correlation between venom lethality and the type of prey consumed, supporting natural selection as one process driving snake venom evolution [1]–[4]. While lethality studies (e.g. LD_50_ experiments) indicate that venom is more likely to kill particular prey, they do not reveal how toxic proteins interact with the physiological systems of the prey to cause incapacitation. Studying toxic activities of venom proteins in prey is of crucial importance in understanding how animal venoms evolve as it is the proteins which are directly subjected to natural selection. Furthermore, the relevance of death as the cessation of all biological functions in prey acquisition is questionable because what determines whether or not the predator gets its meal is disablement of the physiological functions of the prey that would assist escape and or defence. Another problem with lethality studies is that they are often done over an extended time period (e.g. 24 hours) which does not take into account the timeframe in which a particular physiological function is affected [5], and at different venom doses administered different toxins may cause death [6].

The aim of this study is to better understand pathologic processes that affect physiological functions of a rodent organism caused by the venoms of Australian eastern Brown snake *Pseudonaja textilis* populations. This elapid is a prey generalist, feeding on a variety of rodents (including mice and rats ), birds, lizards and frogs [7]. During the attack it wraps coils around its prey [8] and injects its extremely toxic venom with small fangs, which are only 2 to 4 mm long, 2.8 mm on average [9]. *P. textilis* venom contains diverse proteins [10], including venom prothrombin activator pseutarin-C [11], presynaptic neurotoxin textilotoxin [12], [13], type II or ‘long-chain’ [14], [15] and type III ‘short-chain’ α-neurotoxins [16], [17], Kunitz-type serine protease inhibitors textilinins [18], and snake venom phospholipases A_2_ [19]. Several of these proteins have been shown to be lethal when injected into the rodent bloodstream, including *P. textilis* venom prothrombin activator [11], pseudonajatoxin b [14] and short-chain neurotoxins [16].

Studies at the population level are necessary to elucidate processes driving divergence in venom composition and biological activity. Snake venom composition has been found to vary intraspecifically with geographic location [20], [21] and studies have shown correlation between the geographic variation in venom composition and prey type [22], [23]. *Pseudonaja textilis* has a wide distribution in eastern and central Australia and New Guinea, comprising three distinct phylogenetic clades [24], [25]. A study on museum specimens by Shine [7] found that *P. textilis* specimens from Queensland and Northern Territory reach larger body sizes than those from South Australia and Victoria, and that the proportion of endothermic prey (i.e. mammals and birds) increases with body size. Given its wide distribution and variation in body size, *P. textilis* group represents a good model to study processes driving divergence in venom composition and biological activity. Previous studies on geographic variation in composition and bioactivity of *P. textilis* venoms suggested differences in venom prothrombin activator and plasmin inhibitor abundance and activity [26], and differences in textilotoxin D chain and pseudonaja toxin b abundance and post-translational modifications of factor Xa-like heavy chain [10].

In this study, proteomes and biological activities in lab rats (SD) *Rattus norvegicus* of venom samples from Mackay (Queensland) and Barossa (South Australia) *Pseudonaja textilis* specimens are compared. According to the published haplotype clade distribution maps, it can be inferred that Barossa (SA) and Mackay (QLD) populations of *P. textilis* belong to the southeastern and northeastern clades respectively, but this should be verified.

## Materials and Methods

### Venom Samples and Model Animals

Pooled freeze-dried venoms of adult *Pseudonaja textilis* snakes from the Barossa region in the vicinity of Adelaide (South Australia, Australia) and Mackay (Queensland, Australia), *Oxyuranus scutellatus scutellatus* and *O. microlepidotus* were obtained from Venom Supplies, South Australia. *P. textilis* Barossa batches contained venoms pooled from 8 adults. *P. textilis* Mackay batches contained venoms pooled from 2 adults, with venom from 1 additional adult individual obtained and examined subsequently (i.e. a total of 3 adult specimens). All experiments involving rats were done on adult male laboratory Sprague-Dawley rats *Rattus norvegicus*, weighing 300–400 grams. Male 4–10 day old chicks were used for the chick biventer cervicis nerve-muscle preparation.

### Rat Phrenic Nerve – Diaphragm Preparation

Rats were killed by carbon dioxide and exanguination, and the diaphragm with the phrenic nerves was dissected out following Bülbring [27]. A half of the diaphragm muscle with the phrenic nerve was placed in a 50-ml organ bath with physiological salt solution, composed of: 118.4 mM NaCl, 4.7 mM KCl, 1.2 mM MgSO_4_×7 H_2_O, 1.2 mM KH_2_PO_4_, 25.0 mM NaHCO_3_, 11.1 mM D-glucose and 2.5 mM CaCl_2_, bubbled with a 95% O_2_ and 5% CO_2_ gas mixture, and maintained at 37°C. The diaphragm muscle was attached to a FT03 Grass Force-displacement transducer, with tension adjusted to 1 g. The phrenic nerve was threaded through an electrode and stimulated every 10 s by pulses of electric current lasting 0.2 ms at the voltage at which maximum contractions occurred (1–2 V). Pulses were delivered using a Grass S88 stimulator. Prior to experiment, the phrenic nerve response was tested by inducing neuromuscular blockade with 2 µM (+)− tubocurarine chloride (Sigma), after which the agent was washed out.

Venom (10 µg/ml) and was added to the organ bath and left in contact with the tissue until contractions were abolished or for a maximum period of 3 h. The contractions were recorded by ADInstruments MacLab software. The contraction amplitude was calculated by using Max-Min function. The t_95_ value was determined by calculating the time point at which the initial (pre-venom) contraction amplitude was reduced by 95%. The t_95_ values were compared with one-way ANOVA, followed by Holm-Sidak’s multiple comparison test. Statistical analyses and figures in the study were made in GraphPad prism.

### Chick Biventer Cervicis Muscle – Nerve Preparation

This preparation was made following the methods of Ginsborg and Warriner [28] and Harvey et al [29]. Both biventer cervicis muscles with the nerve-enclosing tendons were dissected and transferred to a 5-ml organ bath containing physiological salt solution. The organ bath was bubbled with 95% O_2_ and 5% CO_2_ gas mixture and maintained at 40°C. The resting tension on the tissue was adjusted to 1 g and the nerve-surrounding tendon was electrically stimulated every 10 s with 0.2 ms-pulses. Venom (10 µg/ml) was added to the organ bath, and was washed out after a maximum of 2 h if indirect twitches of the preparation were not abolished. Acetylcholine (1 mM for 30 s), carbachol (0.02 mM for 60 s), and KCl (40 mM for 30 s) were added in the absence of electric nerve stimulation before venom addition and after 2 h of the tissue exposure to the venom. The post-venom chick biventer cervicis muscle responses to acetylcholine were compared between the Mackay and Barossa *P. textilis* venoms with a Student’s t test.

### Venom Procoagulant Activity in Citrated Rat Plasma

Frozen (−80°C) citrated SD rat plasma (anticoagulated with 0.109 M (3.2%) buffered sodium citrate) was thawed in a water bath at 37°C for 5 min. Immediately prior to the assay, thawed plasma (1 ml) was recalcified with CaCl_2_ (4 µmol). The clotting assays were done in 96 well polystyrene clear Greiner Bio-One flat-bottom microplates in a Molecular Devices Versamax microplate reader at a temperature of 37°C. Venom (50 µl of 10 µg/ml) in normal saline was added to 100 µl of recalcified rat plasma, and after an automatic 5 s shake step, absorbance was read every 10 s at the wavelength of 340 nm. The clotting time was recorded as the time of onset of a sharp increase in absorbance, indicating fibrin clot formation (for the general turbidimetric method see [30]). The onset times were obtained with SoftMax Pro 5 microplate data acquisition and analysis software with OD set to 0.02. Clotting times of *Pseudonaja textilis* populations and *Oxyuranus* spp. venoms were analysed with repeated measures one-way ANOVA, followed by Holm-Sidak’s multiple comparison test. *P. textilis* population venom effects at different venom concentrations were tested with repeated measures two-way ANOVA.

### Venom Effects on Circulation of Anaesthetised Rats

Rats were anaesthetised with pentobarbitone sodium (80 mg/kg, i.p., Troy Laboratories Australia Pty Ltd) and placed on a thermostat-controlled heating mat at 37°C. A cannula was inserted in a surgically exposed left carotid artery and connected to a fluid pressure transducer filled with heparinised saline (25 U/ml). The trachea was cannulated and connected to a 7025 Ugo Basile rodent ventilator (approx. 10 ml/kg/stroke, 60 strokes/min). Mechanical ventilation was started before venom or saline injection and maintained throughout the experiment. Freeze-dried venom was dissolved in normal saline, and 0.5 mg in 100 µl saline was injected under the upper dorsal skin of the rat in an imitation of a snake bite, with 4 mm of the hypodermic needle tip inserted. Control rats were injected with an equivalent volume of normal saline. Systemic blood pressure was monitored on a MacLab system (ADInstruments). The mean arterial pressure (MAP) was approximated from systolic (SP) and diastolic pressure (DP) values for 10-second intervals: MAP≈DP+1/3 (SP–DP).

### Venom Effects on Blood Coagulation of Anaesthetised Rats

Collapse of the circulatory system was induced in anesthetised rats by injection of 0.5 mg of venom dissolved in saline (100 µl) under the dorsal skin, and blood was collected from the surgically exposed vena cava. Blood clotting function was assessed by the 20-minute whole blood clotting test (20WBCT). This all-or-none blood coagulability test has been found to be a reliable indicator of low fibrinogen concentration [31], [32]. The test was done by placing approximately 0.5 ml of rat blood in a glass tube (12×75 mm) covered with parafilm and inverting the tube 20 min after. Coagulopathy was indicated if there was no observable clot and the blood fell down on the parafilm.

### Venom Prothrombin Activator Protein-induced Pathology in Anaesthetised Rats

*In vivo* setup was as in the whole venom experiment, with an additional cannula inserted in the right jugular vein and no mechanical ventilation. 20 µg of the size-exclusion HPLC fraction containing the venom prothrombin activator proteins in 100 µl of saline was injected into the jugular vein. Blood was withdrawn from the jugular vein and tested with 20-WBCT.

### Size-exclusion HPLC Venom Profiling

Venom (500 µl of 1 mg/ml) was separated by size-exclusion chromatography on a Superdex G-200 column (13 µm, 10×300 mm, GE Healthcare), with optimum biomolecule separation in the 10–600 kDa range, on a Schimadzu HPLC system (LC-10ATvp pump), and eluted with 0.1 M ammonium acetate buffer (pH adjusted to 6.8), at the flow rate of 0.3 ml/min and protein detection at 280 nm. Manually collected fractions were stored at −80°C, and then freeze-dried. The surface areas under the peaks on the HPLC profile were calculated using GraphPad Prism.

### Isolation of Venom Prothrombin Activator Proteins

Venom prothrombin activator proteins were obtained by collecting the first eluting size-exclusion HPLC fraction without the shoulder containing textilotoxin, using a Superdex G-75 column (GE Healthcare). The flow rate was 0.3 ml/min, elution buffer 0.01 M ammonium acetate (pH = 6.8) and protein detection at 280 nm. The toxins were frozen at −80°C and freeze-dried. The toxin purity was confirmed by LC-MS analysis.

### Mass Spectrometry-liquid Chromatography of Venoms

Venom samples were reconstituted in 100 µl of 20 mM ammonium bicarbonate (NH_4_HCO_3_). 20 µl of each sample was used for digestion with 5 µl of trypsin solution (100 ng trypsin per µl of 1% acetic acid). Acetonitrile was added to 10% v/v and the samples were incubated at 37°C. Tryptic digests were analysed by LC-MS/MS using the QExactive mass spectrometer (Thermo Scientific, Bremen, Germany) coupled online with a RSLC nano HPLC (Ultimate 3000, Thermo Scientific, Bremen, Germany). Samples injected onto a Thermo RSLC pepmap100, 75 µm id, 100 Å pore size, reversed phase nano column with 95% buffer A (0.1% Formic acid) at a flow rate of 300 nl/minute. The peptides were eluted over a 30 min gradient to 70% B (80% acetonitrile, 0.1% formic acid). The eluant was nebulised and ionised using the Thermo nano electrospray source stainless steel emitter with a capillary voltage of 2000 V. Peptides were selected for MS/MS analysis in Full MS/dd-MS^2^ (TopN) mode with the following parameter settings: TopN 10, MSMS AGC target 2e5, 80 ms Max IT, NCE 28 and 2 m/z isolation window. Dynamic exclusion was set to 30 seconds. Data from LC-MS/MS run were exported in Mascot generic file format (*.mgf) using proteowizard 3.0.3631 (open source software, http://proteowizard.sourceforge.net).

### Proteomic Analysis

The mgf. files were analysed in Mascot MS/MS Ion Search using the following parameters: *Enzyme:* Trypsin; *Missed cleavages:* 1; *Taxonomy:* Chordata (Vertebrates and relatives); *Modifications*: none; *Peptide mass tolerance:* ±10 ppm; *Peptide fragment mass tolerance:* ±0.05 Da; *Peptide charge:* +2, +3, +4; *Database:* UniProtKB/Swiss-Prot. The significance threshold was p<0.05. The results were filtered with ion score cut-off set to 0.05 to exclude non-significant matches. Subset proteins were not included in the venome component list since there is no conclusive evidence for their presence.

### Animal Ethics Statement

Experimental procedures were approved by the Monash University Animal Ethics Committee (permits: 2008/05, 2010/35, 2012/008). *In vivo* experiments were done under pentobarbitone sodium anaesthesia. Every effort was made to minimise distress.

## Results

### Rat Diaphragm was Paralysed by the Neurotoxic Effects of *P. textilis* Venoms, but with Large Population Differences in the Paralysis Onset Time

**Figure 1:**
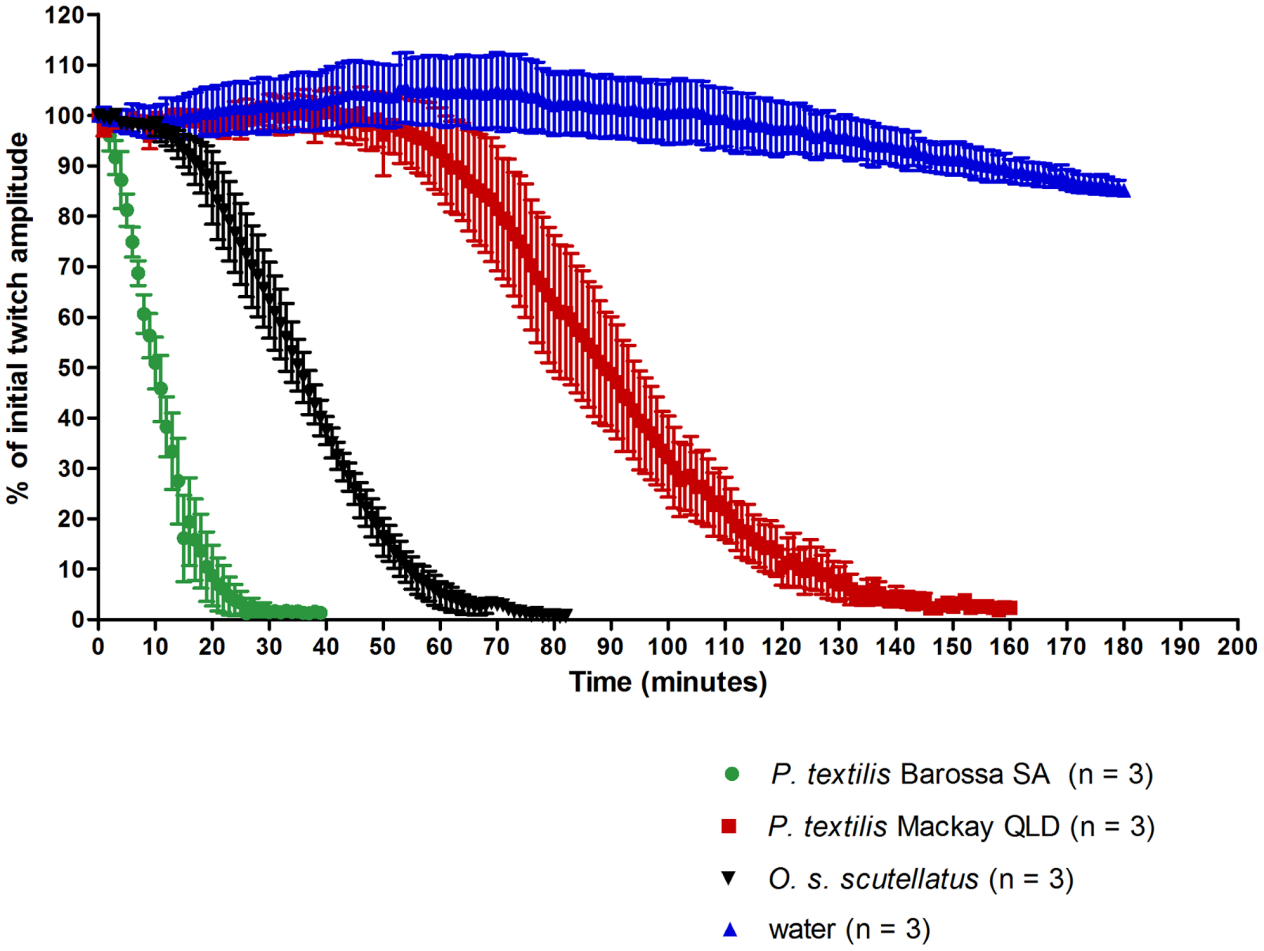
Neurotoxicity in the rat phrenic nerve-diaphragm preparation. 10 µg/ml venom at 37°C (vehicle: water).

Venom of the Barossa *P. textilis* population induced neuromuscular blockade in the phrenic nerve-diaphragm preparation significantly faster than venom from the Mackay population (Fig. 1). The mean time (±1 standard error of the mean) to abolish the initial diaphragm contraction height by 95% (t_95_) at a venom concentration of 10 µg/ml was 21.7±3.5 min by the Barossa/Adelaide (n = 3) and 132.7±6.8 min by the Mackay (n = 3) venom. Interestingly, the difference in neurotoxic activity between the studied populations of *P. textilis* was larger than the difference between *P. textilis* and Australian coastal taipan *Oxyuranus s. scutellatus*, which abolished the diaphragm contractions by 95% in 60.0±3.5 min (n = 3). One-way ANOVA on t_95_ values produced a statistically significant result (p<0.0001), and Holm-Sidak’s multiple comparison test was significant for all comparisons at the significance level (alpha) of 0.05.

### The Population Venoms Differed with Respect to the Contractile Response of the Chick Biventer Cervicis Muscle to Exogenous Acetylcholine

**Figure 2:**
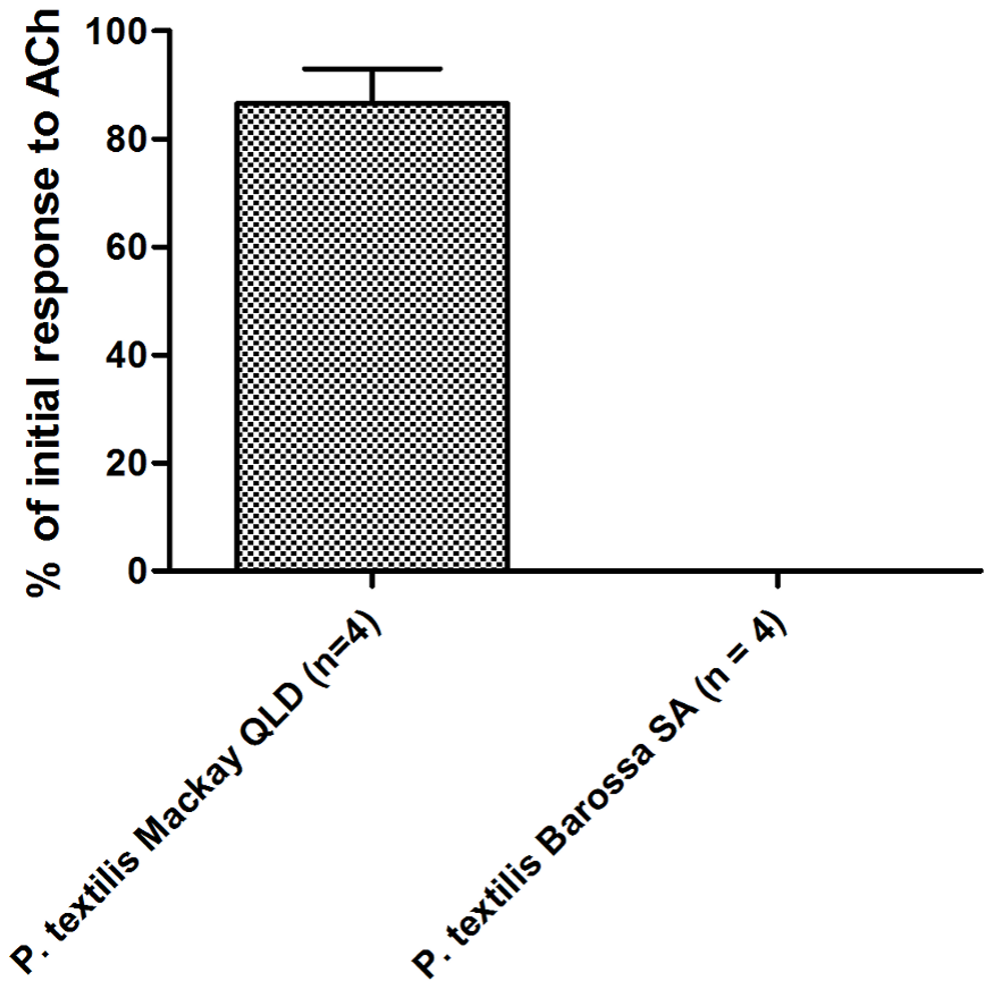
Response to exogenous acetylcholine in the chick biventer cervicis nerve-muscle preparation. 2 hours after exposure to 10 µg/ml venom (vehicle: water).

The contractile response of the chick biventer cervicis muscle to exogenously administered acetylcholine after *P. texti*lis Mackay venom addition was largely preserved, with the mean (± SEM) percentage of initial contractile response of 86.7±6.4%. In contrast, the response after *P. textilis* Barossa venom addition (n = 4) was completely absent (0%) (Fig. 2). The Student’s t test comparing the post-venom chick biventer cervicis muscle responses to acetylcholine between the Mackay and Barossa *P. textilis* population venoms found a statistically significant difference (p<0.0001).

### Both *P. textilis* Population Venoms were Found to be Extremely Procoagulant in Rat Plasma, Mackay (QLD) Population Venom having Higher Potency than that of Barossa (SA)

**Figure 3:**
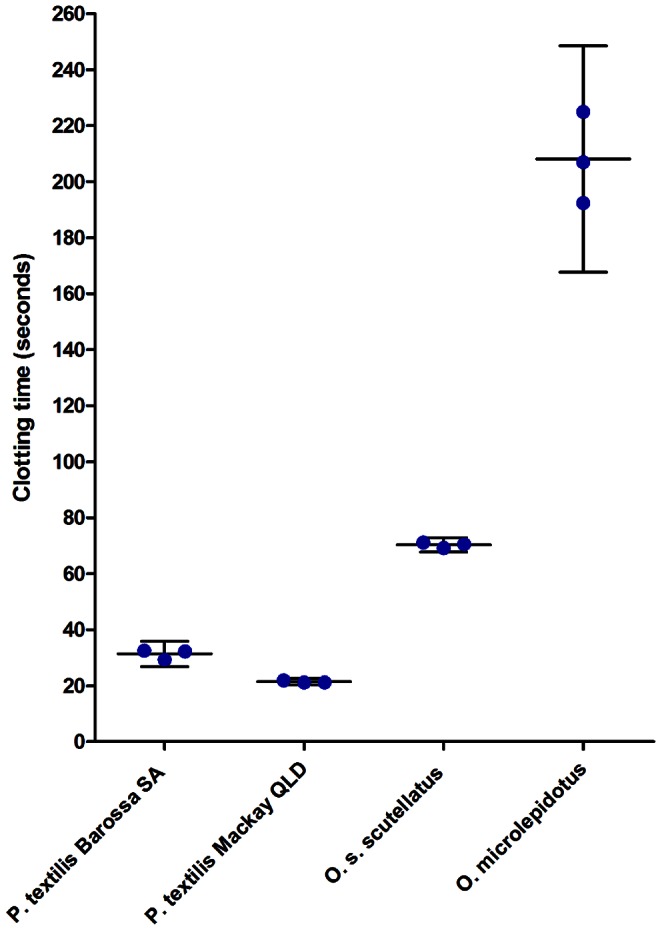
Venom procoagulant activity in rat plasma. 0.5 µg venom (in 50 µl saline) per 100 µl citrated plasma recalcified with 4 µmol CaCl_2_ (means and 95% CI shown).

Venoms of both *P. textilis* populations were found to induce rapid clotting of citrated rat plasma, even at an extremely low dose of 5 µg/100 µl of the plasma, at which the mean time (± SEM) to the onset of clotting was 21.4±0.3 s for the Mackay sample (n = 3) and 31.4±1.0 s for the Barossa (n = 3) population sample. In comparison, venoms of *Oxyuranus* species had lower procoagulant activity than *P. textilis*, with a clotting time of 70.3±0.6 s for *O. scutellatus* (n = 3) and 208.1±9.4 s for *O. microlepidotus* (n = 3) (Fig. 3). A repeated measures one-way ANOVA produced a statistically significant result (p = 0.0026), and a Holm-Sidak multiple comparison test was statistically significant for all species comparisons at the significance level of 0.05. The Mackay *P. textilis* population sample was consistently more potent than the Barossa sample at all concentrations tested: At 50 ng/100 µl of plasma the clotting times were 100.0±4.1 s (n = 3) for venom from the Mackay population and 148.3±4.0 s (n = 3) for the Barossa population, and at 25 ng/100 µl plasma 174.4±9.1 s (n = 3) for Mackay and 263.8±12.3 s (n = 3) for Barossa samples. Repeated measures two-way ANOVA on *P. textilis* population venom effects at different venom concentrations found the population differences statistically significant, with p<0.0001.

### Collapse of the Circulatory System Occurred Instantly Following *P. textilis* Venom Injection in the Rat Dorsum Despite Artificial Respiration

**Figure 4:**
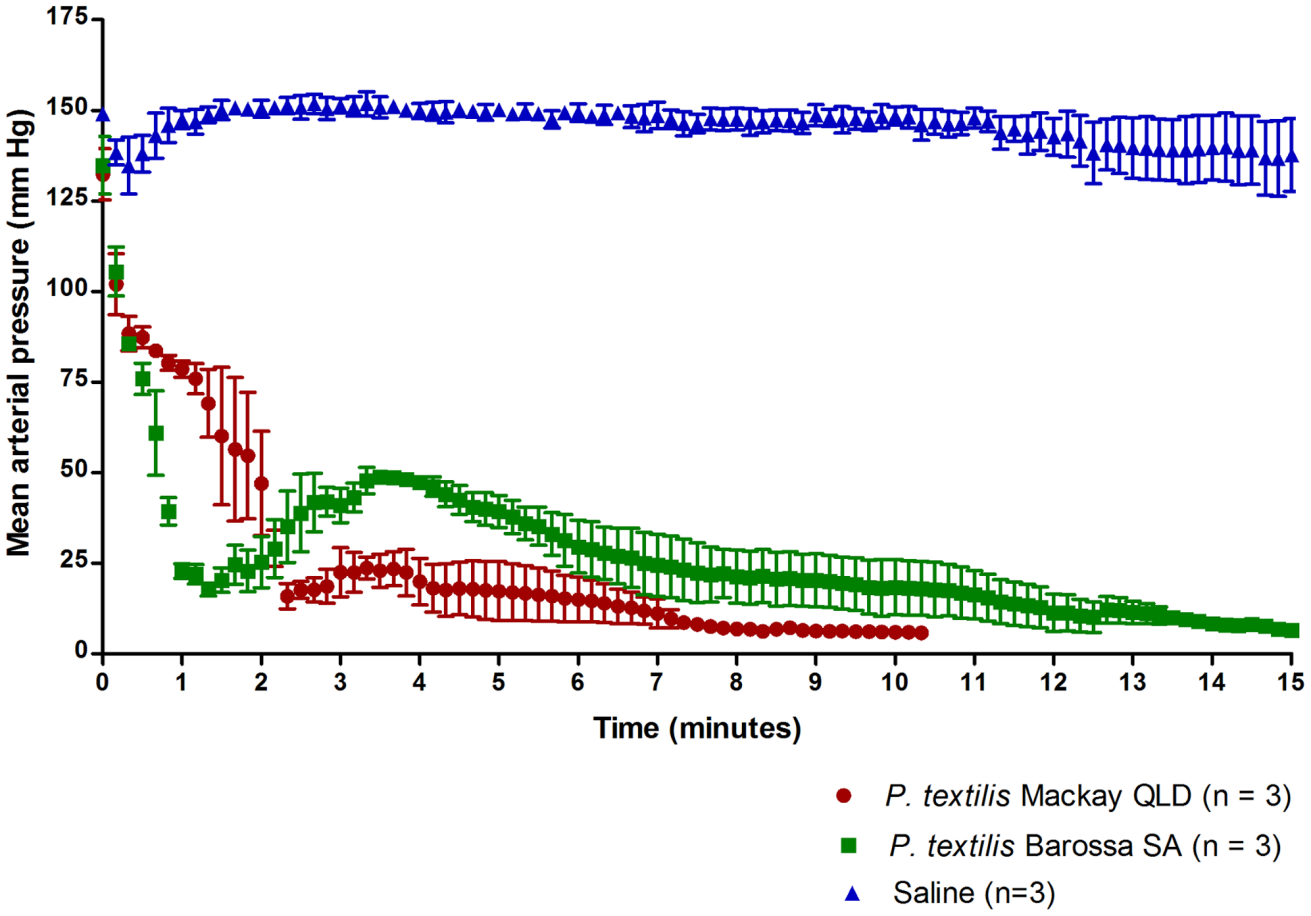
Mean arterial blood pressure in rats injected with 0.5 mg venom under the dorsal skin. Injection volume: 100 µl, vehicle: saline. Rats anaesthetised with 80 mg/kg ip pentobarbitone sodium.

Injection of 0.5 mg of *P. textilis* venom under the dorsal skin of the rat resulted in rapid failure of circulation in 100% of the rats injected (Fig. 4), at sudden drop in the mean arterial pressure below 20 mm Hg in 126.7±13.3 s in Mackay (n = 3) and 76.7±3.3 s in Barossa (n = 3) venom-injected rats. As expected, circulation was unaffected in rats injected with saline only (n = 3).

### Severe Coagulopathy Develops Rapidly Following Injection of *P. textilis* Venom under the Rat Dorsal Skin

Venous blood of rats taken immediately following circulatory collapse induced by venom injection of 0.5 mg of Mackay (n = 3) or Barossa (n = 3) population venoms under the dorsal rat skin was found to be completely incoagulable, whereas that from saline-injected rats (n = 3) clotted normally.

### Injection of *P. textilis* Venom Prothrombin Activator Proteins into the Rat Bloodstream Results in Extremely Rapid Circulatory Failure and Incoagulable Blood

**Figure 5:**
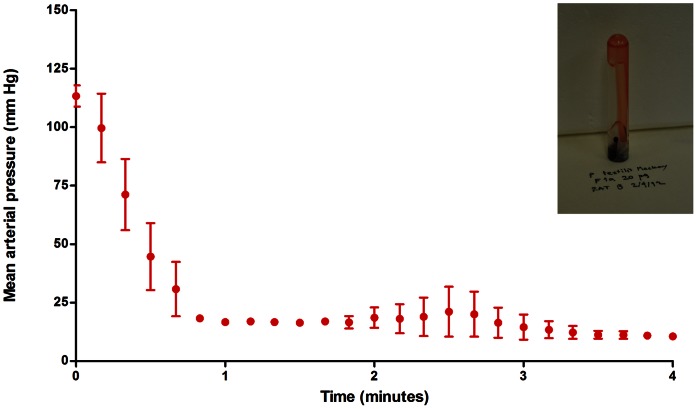
Mean arterial blood pressure in rats injected intravenously *P. textilis* (Mackay) venom prothrombin activator proteins. 20 µg of the HPLC size-exclusion fraction containing proteins matched by LC-MS to Q7SZN0, Q56VR3 and Q1L658 was injected in the jugular vein of pentobarbitone-anaesthesised rats. 20-minute whole blood clotting test confirmed coagulopathic activity of the toxins. Injection volume: 100 µl, vehicle: saline.

Injection of 20 µg of the size-exclusion fraction containing the Mackay *P. textilis* venom proteins matched to the *P. textilis* prothrombin activator pseutarin-C and coagulation factor X isoform 2 (Q7SZN0, Q56VR3, Q1L658) into the jugular vein of the rat caused complete circulatory failure (Fig. 5), with the mean arterial pressure falling below 20 mmHg in 46.7±3.3 s. 20-minute whole blood clotting test confirmed incoagulable blood *in vivo*.

### SE HPLC Revealed Large Differences in the Proportion of the First-eluting Fraction between the *P. textilis* Mackay and Barossa Population Venoms

**Figure 6:**
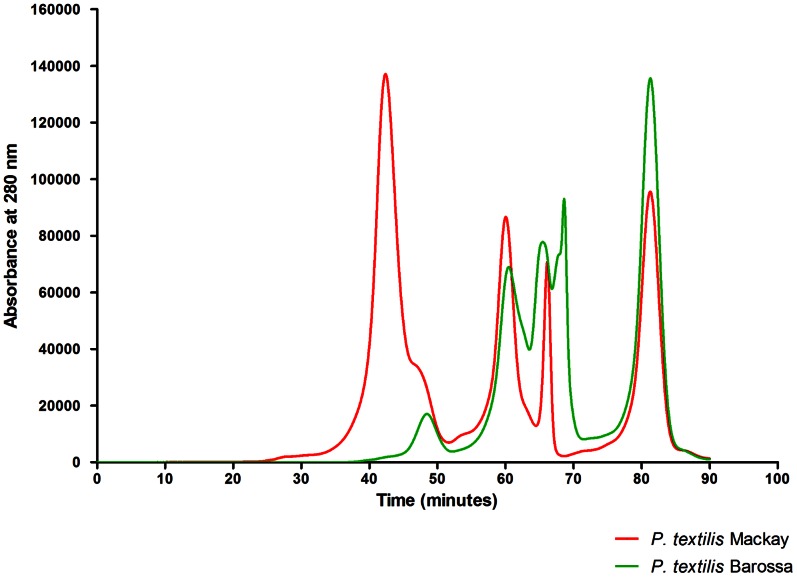
Size-exclusion chromatograms of *Pseudonaja textilis* population venoms. GE Healthcare Superdex G-200 column, 500 µl of 1 mg/ml venom, 0.1 M pH = 6.8 ammonium acetate buffer, 0.3 ml/min.

The highest molecular weight fraction of the Mackay venom comprised 46.6% of the size-exclusion chromatogram. In contrast, this peak comprised only 6.1% in the Barossa sample. Lower molecular weight toxins were more abundant in the venom from the Barossa population (Fig. 6). Ion trap LC-MS analysis of the chromatographic fractions determined that the first eluting fraction contained the venom prothrombin activator proteins and the presynaptic neurotoxin textilotoxin.

### Proteomic Analysis Revealed the Presence of Multiple Coagulation Factor X-like Proteins in the Venoms of *Pseudonaja textilis*

Orbitrap liquid chromatography-mass spectrometry provided a fascinating insight into the venom proteomes of the Australian eastern brown snake populations. Significant protein hits in Mascot are shown in Table 1.

**Table 1:** LC-MS/MS profile of *Pseudonaja textilis* population venom proteomes.

			Mackay QLD *P. textilis*				Barossa SA *P. textilis*			
PROTEIN	ACCESSIONNo.	MASS(Da)	Score	Pep	Seq	Cov (%)	Score	Pep	Seq	Cov (%)
Venom prothrombin activator Pseutarin-Cnon-catalytic subunit *Pseudonaja textilis*	Q7SZN0	165828	31059	981	80	66%	21020	654	65	64%
Venom prothrombin activator Pseutarin-Ccatalytic subunit *P. textilis*	Q56VR3	52181	3037	85	8	24%	2827	82	7	22%
Venom prothrombin activator Oscutarin-Ccatalytic subunit *Oxyuranus scutellatus*	Q58L96	52420					2015	52	5	18%
Coagulation factor X isoform 2 *P. textilis*	Q1L658	51626	1455	56	10	26%	384	13	4	15%
Venom prothrombin activator NotecarinD1 *Notechis scutatus*	P82807	51430					139	12	2	7%
Protease inhibitor textilinin-1 *P. textilis*	Q90WA1	9167					2471	36	6	59%
Protease inhibitor textilinin-2 *P. textilis*	Q90WA0	9173	1818	91	3	39%	2468	34	4	59%
Protease inhibitor textilinin-4 *P. textilis*	Q90W98	8977					88	3	1	16%
Protease inhibitor textilinin-5 *P. textilis*	Q90W97	9025	32	3	1	24%				
Protease inhibitor textilinin-6 *P. textilis*	Q90W96	9054					420	15	1	27%
Phospholipase A_2_ 1 *P. textilis*	Q9W7J4	16833	991	18	2	30%	3046	58	4	58%
Phospholipase A_2_ 2 *P. textilis*	Q9W7J3	17128	1435	55	3	20%	889	40	3	27%
Phospholipase A_2_ textilotoxinA chain *P. textilis*	P23026	13839	63	1	1	8%	44	1	1	8%
Phospholipase A_2_ textilotoxinB chain *P. textilis*	P23027	13789	138	10	3	26%	95	2	2	22%
Phospholipase A_2_ textilotoxinD chain *P. textilis*	P23028	14912	279	10	3	18%	50	2	2	15%
Pseudonajatoxin b homolog*P. textilis*	Q9W7J5	11262					5276	174	7	66%
Pseudonajatoxin b *P. textilis*	P13495	7756					2943	84	4	66%
Long neurotoxin 1 P. textilis	A8HDK6	9875					130	4	1	14%
Short neurotoxin 1/5 *P. textilis*	Q9W7K2	8700					75	3	1	39%
Short neurotoxin 2 *P. textilis*	Q9W7K1	8590					8590	77	3	67%
Short neurotoxin 3 *P. textilis*	Q9W7K0	8576					259	8	3	67%
Short neurotoxin 6 *P. textilis*	Q9W7J7	8564					451	24	1	21%
Short neurotoxin 7 *P. textilis*	Q9W7J6	8880	89	2	1	17%	586	26	2	37%
Pseudechetoxin-like protein*P. textilis*	Q3SB05	26412					228	5	3	22%
C-type lectin BFL-1*Bungarus fasciatus*	Q90WI8	18626					58	3	2	9%

Notes: Mascot protein family search results for each population venom: score, peptide matches (Pep), sequences (Seq) and percent sequence coverage (Cov%). Accession no. = UniProtKB/Swiss-Prot database entry.

Venom prothrombin activator pseutarin-C, with its constituent catalytic (Q56VR3) and non-catalytic (Q7SZN0) subunits, was found in the venom samples of both Barossa and Mackay *P. textilis* populations. Interestingly, the presence of two distinct protein members of the coagulation factor X-like protein family in the venom sample of Mackay *P. textilis* population was revealed. The protein matched to the catalytic subunit of the venom prothrombin activator complex pseutarin-C (*Pseudonaja textilis*) had 6 significant unique peptide sequences, whereas the other protein matching to the coagulation factor X isoform 2 (Q1L658) of *Pseudonaja textilis* had 8 significant unique peptide sequences. Only 2 of the detected peptide sequences were shared by the two proteins, providing strong support for the existence of at least two coagulation factor-X like proteins in the venome of this population (Table S1). The match to *P. textilis* coagulation factor X isoform 2, known to be produced in the liver tissue, is strongly supported, which suggests that the gene coding for this protein or its close homologue may also be expressed in the venom gland.

In the venom sample from the Barossa population, 4 significant peptide sequences were unique to the protein matched to venom prothrombin activator pseutarin-C catalytic subunit, 2 sequences unique to the coagulation factor X isoform 2 of *P. textilis*, and a single significant sequence each unique to venom prothrombin activator oscutarin-C catalytic subunit of *O. scutellatus* (Q58L96) and notecarin-D1 of *N. scutatus* (P82807), confirming presence of multiple coagulation factor X-like proteins in the venoms of this population (Table S2).

### Numerous Postsynaptic α-neurotoxins were Found in *P.* *textilis* Barossa (SA) Venom Whereas they were Largely Absent in the Venom Samples from Mackay (QLD)

LC-MS analysis of the venom sample from Barossa population of *P. textilis* revealed a diverse assemblage of postsynaptic neurotoxins, including ‘long chain’ of the type II (Q9W7J5, P13495 and A8HDK6) and type III (Q9W7K2, Q9W7K1, Q9W7J7, Q9W7J6) alpha three-finger neurotoxins. Interestingly, only a single postsynaptic neurotoxin (Q9W7J6) from the α-neurotoxin type III group was found in Mackay venom sample.

Phospholipase A_2_ subunits of the presynaptic neurotoxin textilotoxin (P23026, P23027, P23028) were found in both populations.

Other noted differences include detection of Pseudechetoxin-like protein (Q3SB05) and a protein with sequence similarity to C-type lectin BFL1 of *Bungarus fasciatus* (Q90WI8) solely in Barossa *P. textilis* venom sample. Different forms of protease inhibitors textililins were found in the venoms of both populations (Table 1).

## Discussion

Venoms from the South Australian and Queensland *P. textilis* populations injected in live rats were found to cause rapid collapse of the circulatory system. Several findings in this study support the role of procoagulant toxins in rapid rodent prey incapacitation: (i) circulatory collapse resulting from intravenous injection of *P. textilis* (Mackay) purified venom prothrombin activators in the rat, including proteins matched to pseutarin-C non-catalytic and catalytic subunits and coagulation factor X isoform 2, (ii) high procoagulant venom activity in rat plasma, and (iii) instant collapse of the circulatory system following injection of venom under the dorsal skin despite artificial respiration. It is remarkable that these proteins have evolved such toxicity to incapacitate and kill endothermic prey as large as a rat in an instant. High procoagulant *P. textilis* venom activity in rat plasma found in this study and histological sections of lung tissue of mice injected i.v. with *Oxyuranus scutellatus* venom showing the presence of thrombi in the study by Herrera et al [6] suggest that thrombosis should be examined as a possible mechanism responsible for rapid collapse of the rodent circulatory system in envenomations with procoagulant venoms of *Pseudonaja* and *Oxyuranus* species.

Venom proteins diversify by gene duplication and subsequent divergence in structure and function [33], which is consistent with multiple coagulation factor X-like proteins found in the venomes of *P. textilis* populations. In addition to venom prothrombin activator pseutarin-C, a significant protein match is to the *P. textilis* coagulation factor X isoform 2, which is particularly well supported for Mackay *P. textilis* venom. Interestingly, the real-time polymerase chain reaction (PCR) study of tissue-specific expression by Reza et al [34] found *P. textilis* coagulation factor X isoform 2 gene to be expressed in the liver, but not in the venom glad.

Venoms of both populations were neurotoxic, causing complete inhibition of the rat diaphragm contractions in the isolated phrenic nerve-diaphragm preparation, but the times to blockade differed significantly. A quicker paralytic effect of Barossa *P. textilis* venom may likely result from the activity of diverse postsynaptic α-neurotoxins, which, with exception of one type III α-neurotoxin, were not found in venom samples from the Mackay population. This hypothesis is supported by the studies in nerve-muscle preparations which showed that the isolated presynaptic PLA_2_ neurotoxins of *P. textilis* (textilotoxin) and *O. microlepidotus* (paradoxin) induce significantly slower onset of neuromuscular blockade than the whole venoms of these species, even at high concentrations of the presynaptic neurotoxins [35], [36]. A study of abundance and bioactivity of the presynaptic PLA_2_ neurotoxins in each population should be carried out to exclude any possibility of the quicker effect observed being due to the abundance or population-specific activity of these neurotoxins. There was no response to exogenously administered acetylcholine in the chick biventer cervicis muscle exposed to the Barossa *P. textilis* venom, which could result from postjunctional blockade of acetylcholine receptors by the postsynaptic neurotoxins (see [29]), but myotoxicity should be assessed at the population level as well. Interestingly, the venom proteome of the Barossa population contains diverse postsynaptic α-neurotoxins, which are mostly lacking in venom samples from the Mackay population. The adaptive roles of these neurotoxins in prey incapacitation are unknown, but due to skeletal muscle paralytic activity, they could potentially be advantageous in acquisition of ectothermic prey.

Venoms of both Mackay and Barossa populations of *P. textilis* have extremely potent procoagulant venom activity in rat plasma, but the Queensland venom appears to have a larger concentration of venom prothrombin activator proteins and is more procoagulant. The procoagulant activities of *O. scutellatus* and *O. microlepidotus* venoms in rat plasma were significantly lower than those of *P. textilis* populations. This finding is consistent with the SDS-PAGE profile of *Pseudonaja* spp. and *Oxyuranus* spp. venoms, with *P. textilis* showing a much denser band than those of *O. scutellatus* and *O. microlepidotus*, indicating a higher concentration of venom prothrombin activator [11]. Despite the venom being less procoagulant than that of *P. textilis* in rodent plasma, *O. scutellatus* is known to deliver high yields of venom in multiple quick bites, and thus large amounts of the venom prothrombin activator and neurotoxic proteins. Different venom yield potentials of *P. textilis* populations and *Oxyuranus* species are demonstrated in the study by Mirtschin et al [37]. A recent study [6] reports that mice injected i.v. with a high dose of whole venom of *O. scutellatus* died sooner than those injected with the same dose of the purified presynaptic PLA_2_ neurotoxin (taipoxin), suggesting that a role of procoagulant toxins in rapid rodent prey incapacitation should be considered for this species.

Physiological responses of the readily available lab SD (Sprague-Dawley) rat *Rattus norvegicus* to injected venom proteins can give us a good insight into pathological processes that occur in envenomed rodent prey, but because susceptibility to venom proteins may differ, when feasible, studies of venom toxicity should be conducted on wild prey. The number of Mackay individuals from which the venom was pooled was small, but the venom from the recently captured third individual showed the same bioactivity and proteomic profile as the venom samples pooled from the two previously collected individuals, supporting the finding of population differences in venom composition and bioactivities in *P. textilis*. Further support is from observations of the venom sample from another Queensland population of *P. textilis* from the Gold Coast lacking significant postsynaptic neurotoxicity as well (unpublished data). Flight et al [26] reported that the venom of the Gold Coast population has higher procoagulant activity in human plasma and a higher first size-exclusion chromatographic peak than that of the South Australian population. This suggests that the venome and bioactivity profile of the Mackay *P. textilis* venom described in this study is likely widespread in populations of the northeastern clade of *P. textilis*. The study should be expanded to include a larger number of individuals and sampling from more localities. Importantly, additional mass spectrometry techniques should be used to study the venom proteomes and potentially detect toxins which may not have been detected with the single LC-MS technique used in this study. In conclusion, high divergence in venom composition and bioactivity of South Australian and Queensland populations of *P. textilis* is revealed, and multiple experiments support the hypothesis of procoagulant toxins causing rapid collapse of the circulatory system in envenomed rodent prey.

## Supporting Information

Table S1 **Coagulation factor X-like protein family in** ***P. textilis*** **Mackay (Queensland, Australia) venom sample.** Software: Mascot; Database: UniProtKB/Swiss-Prot. Proteins are denoted with Roman numerals: I = venom prothrombin activator pseutarin-C catalytic subunit *Pseudonaja textilis* (Q56VR3); II = coagulation factor X isoform 2 *P. textilis* (Q1L658). X denotes presence of a peptide in a particular protein.
(PDF)

Table S2 **Coagulation factor X-like protein family in** ***P. textilis*** **Barossa (South Australia, Australia) venom sample.** Software: Mascot; Database: UniProtKB/Swiss-Prot. Proteins are denoted with Roman numerals: I = venom prothrombin activator pseutarin-C catalytic subunit *Pseudonaja textilis* (Q56VR3); II = venom prothrombin activator oscutarin-C catalytic subutnit *Oxyuranus scutellatus* (Q58L96); III = coagulation factor X isoform 2 *P. textilis* (Q1L658); IV = venom prothrombin activator notecarin-D1 Notechis scutatus (P82807). X denotes presence of a peptide in a particular protein.
(PDF)
