# Supplementary material for: Population Divergence in Venom Bioactivities of Elapid Snake Pseudonaja textilis: Role of Procoagulant Proteins in Rapid Rodent Prey Incapacitation
Source: PLoS One. 2013 May 14;8(5):e63988. doi: 10.1371/journal.pone.0063988 (PMC3653870; doi:10.1371/journal.pone.0063988)
Supplement: Table S1 — Coagulation factor X-like protein family in P. textilis Mackay (Queensland, Australia) venom sample. Software: Mascot; Database: UniProtKB/Swiss-Prot. Proteins are denoted with Roman numerals: I = venom prothrombin activator pseutarin-C catalytic subunit Pseudonaja textilis (Q56VR3); II = coagulation factor X isoform 2 P. textilis (Q1L658). X denotes presence of a peptide in a particular protein. (PDF) [file pone.0063988.s001.pdf]

Table S1. Coagulation factor X-like protein family in *P. textilis* Mackay (Queensland, Australia) venom sample.

Peptide	m/z (exp)	Mr (exp)	z	Mr (calc)	Score	Expect	I	II
IVNGMDCK	440.2086	878.4027	2	878.399	25	0.042	X	X
IETGPLLSVDK	586.33	1170.6454	2	1170.6496	43	0.00057		X
QDFGIVSGFGR	591.7991	1181.5837	2	1181.5829	56	1.8e-05		X
SRIETGPLLSVDK	472.27	1413.7882	3	1413.7827	35	0.0049		X
LGECPWQAALVDDK	772.87	1543.7254	2	1543.7341	80	1.5e-07	X	
LGECPWQAALVDEK	779.88	1557.7454	2	1557.7497	42	0.00088		X
QDFGIVSGFGGIFER	814.9073	1627.8	2	1627.7995	67	6.9e-07	X	
QDFGIVSGFGGIFER	543.6074	1627.8003	3	1627.7995	45	7.2e-05	X	
DACQGDSGGPHTTVYR	555.24	1662.6982	3	1662.7057	81	1.9e-08		X
LGECPWQAALVDDKK	558.283	1671.8272	3	1671.829	67	4.5e-06	X	
DTHFITGIVSSGEGCAR	875.42	1748.8254	2	1748.8152	46	0.00034		X
AETGPLLSVDKVYVHK	439.75	1754.9709	4	1754.9567	48	0.00013	X	
DTHFITGIVSWGEGCAR	924.94	1847.8654	2	1847.8625	40	0.0013	X	
DTHFITGIVSWGEGCAR	616.9627	1847.8662	3	1847.8625	50	0.00013	X	
FDLAAYDYDIAIIQMK	945.4712	1888.9279	2	1888.9281	76	3.8e-07		X
FDLVSYDYDIAIIQMK	645.32	1932.9382	3	1932.9543	56	4.2e-05	X	
FDLVSYDYDIAIIQMK	967.485	1932.9555	2	1932.9543	86	4.1e-08	X	
IVNGMDCKLGECPWQAALVDEK	807.06	2418.1582	3	2418.1382	29	0.012		X
TPIQFSENVVPACLPTADFANQVLMK	945.1445	2832.4116	3	2832.419	51	7.2e-05	X	X

Notes: Software: Mascot; Database: UniProtKB/Swiss-Prot. Proteins are denoted with Roman numerals: I = venom prothrombin activator pseutarin-C catalytic subunit *Pseudonaja textilis* (Q56VR3); II = coagulation factor X isoform 2 *P. textilis* (Q1L658). X denotes presence of a peptide in a particular protein.
