# Supplementary material for: Population Divergence in Venom Bioactivities of Elapid Snake Pseudonaja textilis: Role of Procoagulant Proteins in Rapid Rodent Prey Incapacitation
Source: PLoS One. 2013 May 14;8(5):e63988. doi: 10.1371/journal.pone.0063988 (PMC3653870; doi:10.1371/journal.pone.0063988)
Supplement: Table S2 — Coagulation factor X-like protein family in P. textilis Barossa (South Australia, Australia) venom sample. Software: Mascot; Database: UniProtKB/Swiss-Prot. Proteins are denoted with Roman numerals: I = venom prothrombin activator pseutarin-C catalytic subunit Pseudonaja textilis (Q56VR3); II = venom prothrombin activator oscutarin-C catalytic subutnit Oxyuranus scutellatus (Q58L96); III = coagulation factor X isoform 2 P. textilis (Q1L658); IV = venom prothrombin activator notecarin-D1 Notechis scutatus (P82807). X denotes presence of a peptide in a particular protein. (PDF) [file pone.0063988.s002.pdf]

Table S2. Coagulation factor X-like protein family in *P. textilis* Barossa (South Australia, Australia) venom sample.

Peptide	m/z (exp)	Mr (exp)	z	Mr (calc)	Score	Expect	I	II	III	IV
YGVYTK	365.69	729.3654	2	729.3697	36	0.002		X		X
LGECPWQAALVDDK	772.87	1543.7254	2	1543.7341	74	6.5e-07	X			
LGECPWQAALVDEK	779.88	1557.7454	2	1557.7497	28	0.027			X	
QDFGIVSGFGGIFER	814.9062	1627.7979	2	1627.7995	74	1.5e-07	X			
LGECPWQAALVDDKK	558.2832	1671.8277	3	1671.829	43	0.001	X			
LGECPWQAALVDDKK	836.9212	1671.8278	2	1671.829	66	5e-06	X			
DTHFITGIVSSGEGCAR	875.42	1748.8254	2	1748.8152	43	0.00067			X	
DGIGSYTCTCLSGYEGK	877.3715	1752.7285	2	1752.7335	95	8.3e-10	X	X	X	
DTHFITGIVSWGEGCAR	616.9606	1847.8601	3	1847.8625	49	0.00012	X			
DTHFITGIVSWGEGCAR	924.94	1847.8654	2	1847.8625	72	6.8e-07	X			
FDLVSYDYDIAIIQMK	967.4854	1932.9563	2	1932.9543	82	1.1e-07	X	X		
DTHFITGIVSWGEGCAQTGK	703.0008	2105.9807	3	2105.9841	64	3.7e-06		X		
DTHFITGIVSWGEGCAQTGK	1054.0009	2105.9873	2	2105.9841	72	6.5e-07		X		
TPIQFSENVVPACLPTADFANQVLMK	1417.2155	2832.4164	2	2832.419	73	4.7e-07	X	X	X	
TPIQFSENVVPACLPTADFANQVLMK	945.1471	2832.4194	3	2832.419	52	6.6e-05	X	X	X	
TPIQFSENVVPACLPTADFANEVLMK	945.48	2833.4182	3	2833.403	48	0.00014				X
TPIQFSENVVPACLPTADFANEVLMK	1417.72	2833.4254	2	2833.403	47	0.0002				X

Notes: Software: Mascot; Database: UniProtKB/Swiss-Prot. Proteins are denoted with Roman numerals: I = venom prothrombin activator pseutarin-C catalytic subunit *Pseudonaja textilis* (Q56VR3); II = venom prothrombin activator oscutarin-C catalytic subutnit *Oxyuranus scutellatus* (Q58L96); III = coagulation factor X isoform 2 *P. textilis* (Q1L658); IV = venom prothrombin activator notecarin-D1 Notechis scutatus (P82807). X denotes presence of a peptide in a particular protein.
